# Depressive symptoms during pregnancy and after birth in women living in Sweden who received treatments for fear of birth

**DOI:** 10.1007/s00737-022-01213-z

**Published:** 2022-02-21

**Authors:** Ingegerd Hildingsson, Christine Rubertsson

**Affiliations:** 1 Department of Women’s and Children’s Health, Uppsala University, Uppsala, Sweden; 2 Department of Health Science, Lund University, Lund, Sweden

**Keywords:** Depressive symptoms, Fear of birth, Pregnancy, Postpartum, EPDS

## Abstract

The aim of this study was to investigate the prevalence of depressive symptoms and associated factors in women who underwent treatments for fear of birth; internet-based cognitive therapy, counseling with midwives, continuity with a known midwife or standard care. A secondary analysis was performed using data collected from four samples of women identified with fear of birth and receiving treatment with different methods. A questionnaire was used to collect data in mid-pregnancy and at follow-up 2 months after birth. Depressive symptoms were assessed using the Edinburgh Postnatal Depressive Scale. In mid-pregnancy, 32% of the 422 women with fear of birth also reported a co-morbidity with depressive symptoms. At postpartum follow-up, 19% reported depressive symptoms 2 months after birth, and 12% showed continued or recurrent depressive symptoms identified both during pregnancy and postpartum. A history of mental health problems was the strongest risk factor for presenting with depressive symptoms. None of the treatment options in this study was superior in reducing depressive symptoms. This study showed a significant co-morbidity and overlap between fear of birth and depressive symptoms. Screening for depressive symptoms and fear of birth during pregnancy is important to identify women at risk and offer specific treatment.

## Introduction

Fear of birth and depressive symptoms each affect 10–20% of all pregnant women. There is, however, sparse knowledge about the impact of different treatment options for fear of birth when depressive symptoms also are prevalent during pregnancy and after birth.

There is no universal definition of fear of birth and several methods are used to identify women with fear of birth during pregnancy (Nilsson et al. 2018). Despite the lack of clear definition, fear of birth has been acknowledged in Sweden since the mid-1990s and women who present with fear of birth (self-reported or through a screening procedure) are currently offered counseling with midwives in all Swedish hospitals (Larsson et al. 2016). Women in Sweden with fear of birth are usually referred to a special counseling clinic by the antenatal midwife, who is the primary caregiver during pregnancy. Usually, hospital-based midwives provide the counseling for women with fear of birth. If women request a cesarean section, due to fear of birth, they are usually referred to an obstetrician. Psychologists or social counselors are sometimes also working with women with fear of birth. Such specialist support for women with fear of birth differ in terms of organization of care and resources available (Larsson et al. 2016). Women value the extra support, despite the limited effect on the level of fear and request for cesarean section, which is often prominent in women with fear of birth (Larsson et al. 2017).

Research on fear of birth is growing worldwide and international studies have shown that fear of birth overall affects around 14% of women (O'Connell et al. 2017) and 10–20% in Swedish women (Hildingsson et al. 2017; Nilsson et al. 2018). Fear of birth is important to identify as it could result in negative birth experience and PTSD as concluded in a meta-analysis of 50 scientific papers (Ayers et al. 2016). Several attempts have been introduced to reduce the fear of birth. In a recent systematic review of 18 studies, the authors concluded that some therapies might be effective, such as prenatal education, psychoeducation, and counseling. The result also showed that treatment such as cognitive behavioral therapy would benefit from more research (Aguliera-Martin et al. 2021).

Studies have found that women with fear of birth often present with depressive symptoms (Wikman et al. 2020; Lilliecreutz et al. 2021) or show more signs of psychological strain than generally expected in pregnant women (Rondung et al. 2018a). Some background characteristics are common both in women with fear of birth and those with depressive symptoms, such as younger age, low level of education, and not being native born (Dennis and Hodnett 2007; Santos et al. 2017; Dencker et al. 2019).

Previous international and national studies have shown a prevalence of depressive symptoms during pregnancy in 7–16% of women (Josefsson et al. 2001; Bennett et al. 2004; Rubertsson et al. 2005a; Okagbue et al. 2019). During postpartum, a prevalence of 9–12% is reported (Woody et al. 2017). The overall perinatal prevalence of *major depression* has been estimated to 9%, in a systematic multicenter review comprising 7315 participants (Lyubenova et al. 2020).

The prevalence of depressive symptoms during pregnancy for women in Sweden has been estimated to 13% (EPDS > 13) in a national sample of pregnant women (Rubertsson et al. 2003). Two months after birth, the overall prevalence of depressive symptoms was 12% (EPDS > 12) (Rubertsson et al. 2005b). One group presented with depressive symptoms (EPDS > 12) only during pregnancy (8.9%), one group only 2 months after birth (6.5%), and one group showed depressive symptoms both during pregnancy and after birth (5.8%) (Rubertsson et al. 2005b). A recent Swedish population-based study of 2466 women showed similar results. Five trajectories of depressive symptoms resulted in one healthy group (60.6%), one group with depressive symptoms only during pregnancy (8.5%), 10.9% early postnatal depression (6 weeks after birth), 5.4% late postnatal depression (6 months after birth), and sustained depression (14.6%) (Wikman et al. 2020). Swedish health authorities do not yet recommend screening for depressive symptoms during pregnancy. However, recommendations are provided regarding certain questions the antenatal midwife should ask (SFOG & SBF 2016), and some health regions have introduced screening for depressive symptoms together with assessment of fear of birth (Region Stockholm 2020). National recommendations suggest that cognitive behavioral therapy might help to treat depressive symptoms during the perinatal period, but too few studies are available to suggest best treatment for fear of birth (SBU 2021).

### Aim

The aim of this study was to investigate the prevalence of depressive symptoms and associated factors in women who underwent treatments for fear of birth; internet-based cognitive therapy, counseling with midwives, continuity with a known midwife or standard care.

## Methods

### Design

This is a secondary analysis of a combined sample of women with fear of birth, who underwent four different treatments for their fear, and was investigated using similar questionnaires in mid-pregnancy and 2 months after birth.

### Setting

The studies were conducted in Sweden in one large city (projects 1, 2, and 4), four medium-sized cities (projects 1 and 2), and one small town (project 3) in the middle and northern parts of the country. All women were enrolled for antenatal care in their community, following the national program for antenatal care (SFOG & SBF 2016). Midwives are the primary caregivers in antenatal care in Sweden, where women are usually cared for by the same midwife during approximately nine visits, and during an uncomplicated pregnancy, there is no visit to a physician. All hospitals in Sweden offer counseling for fear of birth with specially trained midwives. In some antenatal clinics, women are offered a screening procedure for fear of birth, and in other clinics, women self-report to the midwife and are subsequently referred to counseling teams when needed. At the time of recruitment, none of the antenatal clinics where participants received care during pregnancy had a screening program to identify depressive symptoms.

### Sample

All participants included in the projects presented with fear of birth. The level of fear of birth was assessed using the Fear of Birth Scale (FOBS). The FOBS scale consists of two 100 mm Visual Analog Scales that are summed and averaged to get a score. When filling out the scale study, participants are asked to respond to the question “How do you feel right now about the approaching birth?” and are instructed to place a mark on the two scales which have the anchor words calm/worried and no fear/strong fear. The cut-off point of 60 or more was used to classify women with fear of birth. FOBS has been psychometrically tested and validated against the Wijma Delivery Expectancy Questionnaire (WDEQ), in a large Australian sample (Haines et al. 2015). The correlation between the instruments was strong; (Rho = 0.66, *p* < 0.001). The area under the ROC was 0.89 indicating high sensitivity with a FOBS cut-off point of 54. Sensitivity was 89%, specificity 79%, and Youden index 0.68. Positive predictive value was 85% and negative predictive value 79%. For practical reasons, after discussion with counseling midwives and obstetricians, a cutoff point of FOBS 60 or more is now used in clinical practice. For the combined present sample Cronbach’s alpha was 0.85. The mean FOBS was 73.82 (SD 17.57), range 0–100. An overview of the projects, the treatments, the grouping for the present study, and the context of care is presented in Table 1.

**Table 1:** Overview of the projects

Original projects	Setting	Original sample	Antenatal care	Intrapartum care	Grouping in the present study
1. U-CARE –pregnancy randomized controlled trial Ethics approval: 2013–209	1 large city 2 middle-sized cities in the middle and northern part of Sweden	258 women with fear of birth randomized into internet-based CBT or counseling	All antenatal care provided in the community with a known midwife	Midwives at the labor wards (usually unknown to the women)	A Internet-based CBT (127) B Counseling (131)
2. AuroraPlus Clinical study Ethics approval: 2016–0588	3 middle-sized cities in the middle and northern part of Sweden	77 women with fear of birth who received counseling	All antenatal care provided in the community with a known midwife	Midwives at the labor wards (usually unknown to the women) or intrapartum care by the counseling midwife	B Counseling (57) C Continuity (22)
3. Midwife all the way Clinical study Ethic approval: 2017–120-31	One small rural town and 2 labor wards within 120 km	75 women with fear of birth	Same midwives providing antenatal and intrapartum care	Continuity with a known midwife or standard care	C Continuity with a known midwife during pregnancy and birth (18) D Standard care (no known midwife during labour and birth)(45)
4. Antenatal Feasibility study Ethic approval: 2016–0588	1 large city, labor ward close	10 women with fear of birth	Same midwives providing antenatal and intrapartum care	Continuity with a known midwife or standard care	C Continuity with a known midwife during pregnancy and birth (8) D Standard care (no known midwife during labor and birth) (2)

### Recruitment of participants

Participants for the present study came from four different treatment projects. All women selected for the present study presented with fear of birth.

For project 1, a randomized controlled trial, the recruitment was done stepwise. First, all Swedish-speaking women who came for a routine ultrasound examination during gestational weeks 17–19 filled out a screening questionnaire including FOBS: the Fear of Birth Scale (Haines et al. 2011; Hildingsson et al. 2017). A research midwife telephoned those who scored 60 or above on the FOBS and asked if they were willing to participate in the study. If they consented to participate, they were sent login details to the internet portal, and all questionnaires were completed online. After entering their background data, women were randomized by the portal program either to internet-based cognitive behavioral therapy (iCBT) or counseling with midwives. The iCBT consisted of 8 weekly modules and supervision from a psychologist. Midwifery counseling was based on the woman’s needs, but usually contained 1–3 counseling visits. Details of the process are presented elsewhere (Rondung et al. 2018b).

For project 2, a clinical study, women were referred to the counseling team by the antenatal midwife after screening with FOBS or self-reported fear of birth. They received oral and written information about the study, and if they consented to participate, they filled out the first of three questionnaires, with background data in mid-pregnancy (Hildingsson et al. 2019a). All women received 1–3 counseling visits and in addition, the counseling midwife also provided intrapartum care for the women they met during counseling, when it was possible (if a counseling midwife was present at the hospital when women were admitted). If a counseling midwife was not available, the women received standard intrapartum care with an unknown midwife.

Project 3, a clinical study, contained selected women participating in a continuity of midwifery care project who scored 60 or more on the FOBS scale in mid-pregnancy. Midwives provided all antenatal care in the small town and were on-call for births between 7 a.m. to 11 p.m. Shortly before the project started, the small labor ward in the town had closed, and women had to travel 110–120 km to reach the nearest hospital with a labor ward. The project midwives took care of counseling for fear of birth for most of the women, except for two women with severe fear who needed extra support (Hildingsson et al. 2020a). If a project midwife was not available (if women gave birth outside the on-call hours or midwives’ were on sick-leave), the women received standard intrapartum care with an unknown midwife.

In project 4, a feasibility study, all women who came for their booking visit in early pregnancy at the antenatal clinic were screened for fear of birth, and those who presented with FOBS scores of 60 or more were taken back in gestational week 20 for a new screening procedure. If fear was still present, two of the midwives arranged a schedule with on-call services from 7 a.m. to 11 p.m. for women with a due date in selected periods from November 2016 to April 2017 (Hildingsson et al. 2018). The antenatal midwives focused on women’s fear of birth, through supportive conversations and provided suggestions for coping mechanisms during all visits. If the antenatal midwives were not available at the onset of labor, the women received standard intrapartum care with an unknown midwife.

### Data collection

Data were collected using one questionnaire distributed in mid-pregnancy (second trimester) and one follow-up questionnaire 2 months after birth. Women in project 1 completed all questionnaires online, whereas women in projects 2–4 received the printed questionnaire by post at their home address. The follow-up questionnaire was distributed in the same way, except women in project 3 also had the opportunity to access a web-based questionnaire if they preferred.

*Background data* in the first questionnaire included self-reported information about age (years), parity (primiparas vs multiparas), marital status (cohabiting with partner vs single), level of education (high school or lower vs university education), country of birth (Sweden vs other countries), infertility treatment (yes/no), history of any previous mental health problems (yes/no), birth preference (vaginal birth/cesarean section), and tobacco use (yes no).

*Depressive symptoms* were assessed using the Edinburgh Postnatal Depression Scale (EDPS) (Cox et al. 1987). EPDS has been validated against DSM criteria during pregnancy in a Swedish study; 13 or above was suggested as a valid cutoff to indicate depressive symptoms during pregnancy (Rubertsson et al. 2011). In the questionnaire completed 2 months after birth, EPDS was filled out again, this time with the cutoff point of 12 or more as suggested if used in the postnatal period (Rubertsson et al. 2005b; Wickberg and Hwang 1996). In the EPDS, three questions refer specifically to anxiety and were also reported (Matthey et al. 2013).

Chronbach’s alpha value for the measure during pregnancy was 0.75 and 0.875 after birth. Mean values were 13.72 (SD 5.30, range 0–26 during pregnancy and 11.41 (SD 6.81, range 0–26) after birth.

### Analysis

Descriptive statistics were used to present the background characteristics. Differences between women’s background characteristics and treatment for fear of birth was explored using chi-square test. The continuous EPDS scores over time and between treatment methods were analyzed using a mixed between-within subjects analysis of variance (ANOVA) test, with and without covariates included, and with calculation of mean scores and reduction in mean scores over time (Pallant 2013).

To study co-morbidity, a composite variable was created with the score 0 = no depressive symptoms during pregnancy or after birth (reference category); 1 = depressive symptoms during pregnancy but not after birth; 2 = depressive symptoms after birth but not during pregnancy; and 3 = depressive symptoms on both occasions. Differences between the groups in the composite variable, background factors, and treatment options were calculated using odds ratios with a 95% confidence interval or Fisher’s exact test. A *p*-value < 0.05 was regarded statistically significant. The odds ratios were calculated using a multi-nominal regression analysis in the statistical program SPSS.

## Results

### Depressive symptoms during pregnancy in relation to background characteristics

The total sample consisted of 422 women with fear of birth. Table 2 shows the participants’ background information. The majority of participants were 25–35 years old, living with a partner, born in Sweden, and around half had a university education. There were more primiparas (60%) than multiparas (40%); few participants had had infertility problems, and the percentage of tobacco users was low. Every fourth woman preferred a cesarean section, and around 44% reported a previous history of mental health problems. The proportion of women who presented with depressive symptoms in mid-pregnancy using EPDS with the cutoff of 13 was 32.2%. In total, 190 women (45.0%) received counseling, 127 (30.1%) received iCBT, 58 women received continuity of care (13.7%), and 47 standard care (11.1%).

**Table 2:** Background of the participants

	*Data collected in mid-pregnancy* Participants *n* = 422 *n* (%)	*Data collected two months after birth* Participants *n* = 260 *n* (%)
Age groups		
Younger than 25 years 25–35 years Older than 35 years	52 (12.4) 288 (68.4) 81 (19.2)	21 (8.5) 172 (69.4) 55 (22.2)
Marital status		
Living with a partner Not living with a partner	403 (95.5) 19 (4.5)	241 (96.8) 8 (3.2)
Country of birth		
Sweden Other country	376 (89.1) 46 (10.9)	229 (92.0) 20 (8.0)
Level of education		
High school or lower University education	208 (49.3) 214 (50.7)	113 (45.4) 136 (54.6)
Parity		
Primiparas Multiparas	246 (58.9) 172 (41.1)	148 (60.2) 98 (39.8)
Infertility problems		
Yes No	34 (8.2) 382 (91.8)	22 (8.9) 224 (91.1)
Any previous mental health problems		
Yes No	185 (44.3) 233 (55.7)	110 (44.5) 137 (55.5)
Tobacco use		
Yes No	22 (5.2) 400 (94.8)	13 (5.2) 236 (94.8)
Birth preference		
Vaginal birth Cesarean section	313 (75.2) 103 (24.8)	224 (90.0) 25 (10.0)
Depressive symptoms		In mid pregnancy
EPDS < 13 EPDS 13 or more	286 (67.8) 136 (32.2)	123 (49.4) 126 (50.6)

^*^Numbers might not add to 100% due to internal missing values

**Fig. 1:**
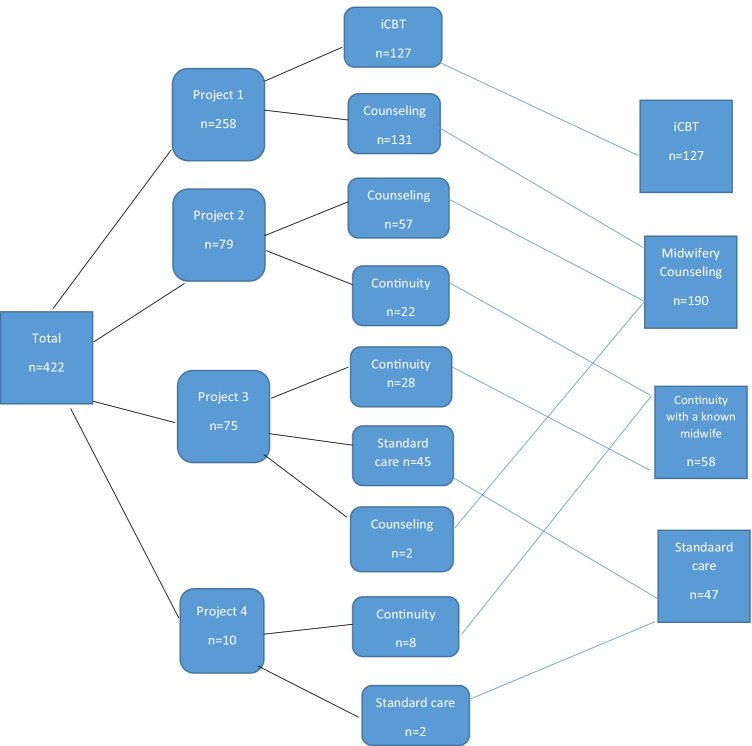
The formation of the treatment groups based on the projects

Figure 1 shows how the treatment groups were formed, based on the four different projects.

Table 3 shows that there were some background differences between women in the treatment groups, in parity, level of education, previous mental health problems, and birth preference.

**Table 3:** Women’s background characteristics in relation to treatment for fear of birth

	Counseling with midwife *n* = 190 *n* (%)	Internet-based CBT *n* = 127 *n* (%)	Continuity of midwifery care *n* = 58 *n* (%)	Standard care *n* = 47 *n* (%)	*p*-value
Age groups					
Younger than 25 years	19 (10.1)	21 (16.5)	4 (6.9)	7 (14.9)	
25–35 years	129 (68.6)	85 (66.9)	40 (69.0)	31 (60.0)	
Older than 35 years	40 (21.3)	21 (16.5)	14 (24.5)	9 (19.1)	0.450
Marital status					
Living with a partner	180 (94.7)	121 (95.3)	58 (100.0)	44 (93.6)	
Not living with a partner	10 (5.3)	6 (4.7)		3 (6.4)	0.335
Country of birth					
Sweden	171 (90.0)	108 (85.0)	51 (87.9)	46 (97.9)	
Other country	19 (10.0)	19 (15.0)	7 (12.1)	1 (2.1)	0.106
Level of education					
High school or lower	91 (47.9)	55 (43.3)	29 (50.0)	33 (70.2)	
University education	99 (52.1)	72 (56.7)	29 (50.0)	14 (29.8)	0.017
Parity					
Primiparas	93 (49.2)	77 (60.6)	34 (60.7)	42 (91.3)	
Multiparas	96 (50.8)	50 (39.4)	22 (39.3)	4 (8.7)	0.000
Infertility problems					
Yes	14 (7.5)	11 (8.7)	5 (8.9)	4 (8.7)	
No	173 (92.5)	116 (91.3)	51 (91.1)	42 (91.3)	0.973
Any previous mental health problems					
Yes	98 (52.1)	54 (42.5)	22 (37.9)	11 (24.4)	
No	90 (47.9)	72 (57.5)	36 (62.1)	34 (75.6)	0.005
Tobacco use					
Yes	9 (4.7)	6 (4.7)	3 (5.2)	4 (8.5)	
No	181 (95.3)	121 (95.3)	55 (94.8)	43(91.5)	0.757
Birth preference					
Vaginal birth	141 (75.4)	84 (66.7)	46 (82.1)	42 (89.4)	
Cesarean section	46 (24.6)	42 (33.3)	10 (17.9)	5 (10.6)	0.010
Depressive symptoms/Anxiety					
EPDS 13 or more	64 (33.7)	47 (32.0)	16 (27.6)	9 (19.1)	0.124
EPDS-Anxiety 6 or more	59 (31.1)	34 (26.8)	16 (27.6)	15 (31.9)	0.823

Almost similar proportions of multiparous and primiparous women had had counseling (49% vs 51%). Around 60% of women who received iCBT and continuity of midwifery care were primiparas, and so were 91% of the women who received standard care. These differences were statistically significant (*p* < 0.000).

In the four treatment groups 50–57% had a university education. The exception was women who had standard care, where only one in three had a high level of education (*p* 0.017). There were also some differences in previous mental health problems (*p* 0.005). The highest proportion of mental health problems was found in women who had counseling (52%), followed by women who had had iCBT (42%), continuity (38%), and the lowest proportion in women who received standard care (24%).

There were also statistically significant differences in birth preferences (*p* 0.010). More than one in three women in the iCBT group preferred a cesarean Section (33%) and so did a quarter of women who received counseling (25%). Among women who had continuity of midwifery care, 18% preferred a cesarean section and so did 11% of women who had standard care. There were no differences between the groups in EPDS-scores, or in the sub scores of anxiety in EPDS.

### Depressive symptoms after birth in relation to background characteristics

The follow-up questionnaire was completed by 260 women (62% of the original sample), but only 256 completed EPDS after birth (Table 2). Women who not completed the follow-up questionnaire were more likely to be born in a country outside Sweden (*p* 0.027) and were more likely to have had EPDS-scores above 13 in mid pregnancy (*p* 0.000). The largest drop-out rate was found in women randomized to iCBT (76%). In total, 19.1% scored 12 or more on EPDS 2 months after birth.

### Changes in depressive symptoms over time

As one of the aims of this study was to explore if there were any changes in depressive symptoms in relation to treatment for fear of birth, we analyzed the four treatment groups separately. In Table 4, the EPDS scores are presented for the whole sample and divided by the treatment groups. When studying each treatment group separately, there was a decrease in the mean EPDS scores for all groups except for women who received iCBT, with the highest mean reduction in women who had continuity of care with a known midwife.

**Table 4:** Depressive symptoms in women with fear of birth in relation to treatment

	All women regardless of treatment *n* = 422 *n* (%)	Internet-based CBT *n* = 127 *n* (%)	Counseling with midwife *n* = 190 *n* (%)	Continuity of midwifery care *n* = 58 *n* (%)	Standard care *n* = 47 *n* (%)
Change over time in mean scores					
Mean EPDS mid pregnancy (SD)	10.22 (5.42)	8.35 (6.15)	9.95 (5.33)	9.37 (5.57)	10.00 (5.31)
Mean EPDS after birth (SD)	7.68 (5.29)	6.67 (3.76)	7.97 (5.27)	7.31 (6.19)	8.06 (5.07)
Reduction in mean scores	2.54	1.68	1.98	2.06	1.94
Effect size	0.131	0.087	0.144	0.108	0.200
* p*-value	0.000	0.101	0.000	0.011	0.002

In the next step of the analysis, a mixed between-within subjects’ ANOVA was conducted to assess the impact of the four treatment options on participants’ EPDS scores across time. There was a statistically significant main effect for time, with a reduction in the mean scores on EPDS for the whole sample (*p* < 0.000), with moderate effect size (partial eta square 0.102), but no difference between the treatment methods (*p* 0.424). The result remained statistically significant when adjusting for covariates (Wilks lambda 0.94, *F* 13,957, *p* 0.000), but the effect size was low and there was still no difference between the treatment methods (*p* 0.873).

### Trajectories of depressive symptoms

Of the 251 women who completed both questionnaires, a composite variable was created. The composite variable was built on the prevalence figures of depressive symptoms from the different time points to get a picture of the trajectories. Altogether, 169 (67.3%) women with fear of birth did not have depressive symptoms at all (reference group), 34 women (13.5%) reported depressive symptoms during pregnancy but not after birth, 18 women had EPDS scores of 12 or more after birth (7.2%); these women did not have depressive symptoms in mid-pregnancy. Finally, 30 women (12.0%) presented with depressive symptoms in pregnancy and after birth. The multi-nominal regression analysis showed that depressive symptoms *only in pregnancy* were associated with a history of mental health problems (*OR* 4.63; 2.04–10.52, *p* < 0.001), and a preference for cesarean section (*OR* 4.10; 1.29–13.05, *p* 0.017). Depressive symptoms *only after birth* were associated with a low level of education (*OR* 3.22; 1.07–9.67, *p* 0.037). Depressive symptoms *both during and after pregnancy* were associated with single status (*OR* 7.19; 1.12–45.95, *p* 0.037), and a history of mental health problems (*OR* 4.99; 2.08–11.99, *p* < 0.001). No other associations were found, either in background characteristics or in treatment for fear of birth.

## Discussion

The main findings of this study were that women with fear of birth presented with fairly high co-morbidity with depressive symptoms during pregnancy. A large proportion had a history of mental health problems, which was the strongest explanatory factor for the composite variable of xx.

High levels of depressive symptoms were found in women with fear of birth, with more than 30% during pregnancy and in nearly 20% of women 2 months after birth. These figures are higher compared to the 10–20% of women in general childbearing populations with depressive symptoms (Bennett et al. 2004; Woody et al. 2017; Okagbue et al. 2019), but similar to a Norwegian study of 1600 women which also found that 32% presented with depressive symptoms and fear of birth (Storksen et al. 2012). We do not know if midwives in antenatal care identified this large number or not. Previous studies have shown that depressive symptoms are common in women with fear of birth, but it is possible that fear of birth is not as stigmatized as mental health problems, and easier to communicate and self-report during pregnancy, as there are counseling options available with additional support (Larsson et al. 2016).

Mental health problems are increasing, especially in young women (Public Health Agency of Sweden 2018). If women feel stigmatized due to their mental ill-health, it can evoke feelings of shame and hinder adequate help. A qualitative study from Ireland showed that barriers to disclosing current or previous mental health problems were related to stigma and shame, lack of continuity of care, and lack of time. Women also mentioned that it was a difficult subject during the emotional time of pregnancy when they were supposed to be happy (Nagle and Farrelly 2018). Assessment of mental health could also be challenging for midwives if clear care pathways and options for referrals are sparse.

Although depressive symptoms decreased over time in this population of women with fear of birth, it is still thought-provoking that Swedish antenatal care misses the opportunity to identify women in need of treatment for depressive symptoms. This might be partly explained by the lack of screening procedures during pregnancy, or organizational issues, such as lack of referral options to perinatal psychologists. There is still a debate in Sweden about whether to screen for depressive symptoms or not, despite evidence from international studies (Avalos et al. 2016; O’Connor et al. 2019). Screening might help health care providers refer women for psychological interventions to reduce the risk of perinatal depressive symptoms, as shown in a systematic review (O’Connor et al. 2019).

Pregnant women in Sweden who suffer from fear of birth have a clearly described care pathway with access to a counseling program for help within the system of antenatal and intrapartum care. In addition to counseling with trained midwives, obstetricians and psychologists are usually available for these women. In some areas, women with depressive symptoms during pregnancy are referred by their midwife to perinatal psychologists, while in other areas, women are referred to primary health care and seen by family physicians and/or psychologists working with a variety of patients (SFOG & SBF 2016).

Counseling for fear of birth has been developed to be an established treatment option in Sweden; however, an additional focus is needed for treatment of depressive symptoms. Access to psychologists, psychiatrists, or family physicians if women present with depressive symptoms is important, for women to get adequate help. Another important treatment might be to further introduce *Listening Visits*, an option suggested as an effective, first-line treatment of maternal depression for mothers with mild to moderately severe depression symptoms, as shown in a recent meta-analysis of six studies (McCabe et al. 2021). Research shows that a majority of women prefer to talk about their feelings with a non-judgmental empathic listener with knowledge about mental health in the post-partum period (Dennis and Chung-Lee 2006).

Other important characteristics associated with depressive symptoms in the present study were not living with a partner and low level of education, factors previously identified in women with depressive symptoms (Dennis and Hodnett 2007; Santos et al. 2017).

When considering the different treatments for fear of birth (iCBT, counseling, continuity, and standard care), the mixed between-within subjects’ ANOVA showed no statistically significant differences between the treatment groups in terms of reduction in EPDS scores. Time seemed to be the factor that reduced depressive symptoms, not treatment for fear of birth. We do not know if women in these treatment groups had received any help for depressive symptoms or if women with fear of birth in the four studies that form the basis of this secondary analysis, presented with totally different reasons behind their fears. Women in the continuity group and those who received standard care comprised a large proportion of women living in a rural area with a long distance to hospital, which might be the main reason for expressing fear of birth (Hildingsson and Larsson 2021). Fear of birth is a concept without clear definitions, and scoring high on the FOBS for these groups could just be a reply to being afraid of giving birth in a car along the road far from hospital. The effectiveness of continuity with a known midwife on levels of fear seems to be promising (Hildingsson et al. 2019a) on women’s birth experience (Hildingsson et al. 2019b, 2020b) and for women who present with depressive symptoms, fear, major worries, and low sense of coherence (Hildingsson and Rubertsson 2021). Still, more research is needed in this area in order to help women experience a positive childbearing period.

The findings can also be viewed in light of the different background characteristics of the women in the treatment groups. The women who received counseling and iCBT showed more similar characteristics, as did those who received continuity of a known midwife and those who received standard care. Differences were found in the levels of education, parity, mental health, and birth preference, factors associated with both depressive symptoms and fear of birth (Dennis and Hodnett 2007; Santos et al. 2017; Dencker et al. 2019). However, women in the latter groups had lower education levels were more often primiparas, less likely to prefer a cesarean section and to report previous mental health problems. Preventing unnecessary cesarean sections is important as cesarean sections could affect both women’s and children’s health negatively, and women in Sweden do not have any legal right to choose an operation without medical indication (Karlström et al. 2010).

We also followed the trajectories of depressive symptoms in this sample comprising women with fear of birth. The proportion of depressive symptoms *only during pregnancy* was 13.5%, nearly double that reported by Denckla et al. (2018) and the more recent population-based study with 8.5% of women from a central part of Sweden having a large proportion of highly educated women (Wikman et al. 2020). Depressive symptoms o*nly after birth* was also higher in the present study compared to the study by Denckla et al. (2018) (7% vs. 4%) but lower than the population-based study (10.9%) (Wikman et al. 2020) and the Swedish national survey of Swedish-speaking women, where 13% scored over the corrected cutoff point of EPDS (Rubertsson et al. 2005b).

What is most noteworthy is the proportion of women classified as having depressive symptoms *both during pregnancy and after birth* (12%). These figures could partly be explained by the co-morbidity with fear of childbirth and the previous suggestion that women with fear of birth carry a high degree of psychological strain (Rondung et al. 2018a). This seems logical in the present study, as a history of mental health problems was found in 44% of the whole sample and was an important explanatory variable for two of the trajectories of depressive symptoms (only during pregnancy and on both occasions). Another important variable to consider is women’s birth preferences, which also was a significant predictor of sustained depressive symptoms. Similarly, a recent UK-based study found that 27% of women with severe fear of birth suffered from depression and 24% from anxiety. Having any of the common mental disorders and fear of birth was found in 45% (Nath et al. 2021). There was, however, no association between severe fear of birth and mode of birth. It is well-known that women with fear of birth are more prone to request a cesarean section (Olieman et al. 2017; Larsson et al. 2017), so the explanatory variable in the present study for women with depressive symptoms during and after pregnancy might mirror the sample characteristics of women with fear of birth. However, the majority of participants (75%) preferred a vaginal birth, so it might be that cesarean section preference is a key question to raise and to identify women with depressive symptoms. A systematic review concluded that women with cesarean section requests reported higher levels of depression during pregnancy but not after birth. The authors concluded that the prospect of an elective cesarean section does not lower the levels of depressive symptoms. Despite this fact, the risk of developing depression or PTSD must be considered if a woman persists in her request after adequate counseling or psychiatric treatment (Olieman et al. 2017). Likewise, Ayers and co-workers concluded from a meta-analysis of 50 scientific papers that depression during and after pregnancy, fear of birth, and having an operative birth (vacuum extraction or cesarean section) were factors associated with developing PTSD and that such risk factors should be assessed in connection with screening procedures (Ayers et al. 2016).

This study is compromised by its observational design when conducting a secondary analysis with a mix of women in the treatment groups. Another important issue to consider is the large drop-out rate, mainly in the iCBT group. iCBT has been recommended as a valuable treatment for depressive symptoms in general populations (Swedish National Board of Health and Welfare 2020) but might be less effective or interesting for pregnant women when compared to face-to-face counseling. Classifying fear of birth by using only two questions (FOBS) could be another limitation. However, FOBS is a validated instrument used in many studies and as a valuable screening instrument in clinical practice in Sweden and in other countries. Women also self-report fear of birth to their antenatal midwife, so their fear of birth is likely to be recognized. Using more extensive instruments is mainly used for research purposes. Notable is also that treatment was not randomly assigned, which is a limitation. The lack of statistical power or heterogeneity might also explain the null differences between treatments. When breaking down the sample into small groups, such as “Continuity of care” and “standard care” groups which are smaller than *n* = 50, the standard deviations of the EPDS scores vary considerably in some comparisons.

## Conclusion

This study showed a significant co-morbidity between fear of birth and depressive symptoms, with previous mental health problems as the most important explanatory factor. When identified with fear of birth, an additional anamnesis or screening for depressive symptoms are suggested. Screening for depressive symptoms and fear of birth during pregnancy is important to identify women at risk and offer specific treatment. None of the treatment options for fear of birth in this study was superior in reducing depressive symptoms, but more research is needed to develop models that consider the physical, emotional, and social aspects of pregnancy and childbirth.

## References

[CR1] Aguilera-Martín Á, Gálvez-Lara M, Blanco-Ruiz M, García-Torres F (2021). Psychological, educational, and alternative interventions for reducing fear of childbirth in pregnant women: A systematic review. J Clin Psychol.

[CR2] Avalos LA, Raine-Bennett T, Chen H, Adams AS, Flanagan T (2016). Improved perinatal depression screening, treatment, and outcomes with a universal obstetric program. Obstet Gynecol.

[CR3] Ayers S, Bond R, Bertullies S, Wijma K (2016). The aetiology of post-traumatic stress following childbirth: a meta-analysis and theoretical framework. Psychol Med.

[CR4] Bennett HA, Einarson A, Taddio A, Koren G, Einarson TR (2004). Prevalence of depression during pregnancy: systematic review. Obstet Gynecol.

[CR5] Cox JPL, Holden JM, Sagovsky R (1987). Detection of postnatal depression. Development of the 10-item Edinburgh Postnatal Depression Scale. Br J Psychiatr.

[CR6] Dencker A, Nilsson C, Begley C, Jangsten E, Mollberg M, Patela H (2019). Causes and outcomes in studies of fear of childbirth: a systematic review. Women Birth.

[CR7] Denckla CA, Mancini AD, Consedine NS, Milanovic SM, Basu A, Seedat S (2018). Distinguishing postpartum and antepartum depressive trajectories in a large population-based cohort: the impact of exposure to adversity and offspring gender. Psychol Med.

[CR8] Dennis C-L, Chung-lee L (2006). (2006) Postpartum depression help-seeking barriers and maternal treatment preferences: a qualitative systematic review. Birth.

[CR9] Dennis C-L, Hodnett E (2007). Psychosocial and psychological interventions for treating postpartum depression. Cochrane Database Syst Rev.

[CR10] Haines H, Pallant JF, Karlström A, Hildingsson I (2011). Cross-cultural comparison of levels of childbirth-related fear in an Australian and Swedish sample. Midwifery.

[CR11] Haines H, Pallant J, Toohill J, Creedy D, Gamble J, Hildingsson I, Fenwick J (2015). Identifying women who are afraid of giving birth: a comparison of the fear of birth scale with the WDEQ-A in a large Australian cohort. Sex Reprod Healthc.

[CR12] Hildingsson I, Haines H, Karlström A, Nystedt A (2017). Presence and process of fear of birth during pregnancy- findings from a longitudinal cohort study. Women Birth.

[CR13] Hildingsson I, Rubertsson C, Karlström A, Haines H (2018). Caseload midwifery for women with fear of birth is a feasible option. Sex Reprod Healthc.

[CR14] Hildingsson I, Karlström A, Rubertsson C, Haines H (2019). Women with fear of childbirth might benefit from having a known midwife during labour. Women Birth.

[CR15] Hildingsson I, Karlström A, Rubertsson C, Haines H (2019). A known midwife can make a difference for women with fear of childbirth- birth outcome and experience of intrapartum care. Sex Reprod Healthc.

[CR16] Hildingsson I, Karlström A, Larsson B (2020). A continuity of care project with two on-call schedules: findings from a rural area in Sweden. Sex Reprod Healthc.

[CR17] Hildingsson I, Karlström A, Larsson B (2020b) Childbirth experience in women participating in a continuity of midwifery care project. Woman Birth [E-pub ahead of print] 10.1016/j.wombi.2020.04.010 10.1016/j.wombi.2020.04.010 32595033

[CR18] Hildingsson I, Rubertsson C (2021) The role of women’s emotional profiles in birth outcome and birth experience. J Psychosom Obset Gynecol: 1–9. [E-pub ahead of print] 10.1080/0167482X.2021.1885026 10.1080/0167482X.2021.1885026 33586598

[CR19] Hildingsson I, Larsson B (2021) Women’s worries during pregnancy; a cross-sectional survey using the Cambridge Worry Scale in a rural area with long distance to hospital. Sex Reprod Healthc [E-pub ahead of print]. 10.1016/j.srhc.2021.100610 10.1016/j.srhc.2021.100610 33706121

[CR20] Josefsson A, Berg G, Nordin C, Sydsjo G (2001). Prevalence of depressive symptoms in late pregnancy and postpartum. Acta Obstet Gynecol Scand.

[CR21] Karlström A, Rådestad I, Eriksson C, Rubertsson C, Nystedt A, Hildingsson I (2010). Cesarean section without medical reason, 1997 to 2006: a Swedish register study. Birth.

[CR22] Larsson B, Karlström A, Rubertsson C, Hildingsson I (2016). Counseling for childbirth fear- a national survey. Sex Reprod Healthc.

[CR23] Larsson B, Karlström A, Rubertsson C, Ternström E, Thomtén J, Segebladh B, Hildingsson I (2017). Birth preference in women undergoing treatment for childbirth fear: a randomised controlled trial. Women Birth.

[CR24] Lilliecreutz C, Josefsson A, Mohammed H, Josefsson A, Sydsjö G (2021). Mental disorders and risk factors among pregnant women with depressive symptoms in Sweden—a case-control study. Acta Obstet Gynecol Scand.

[CR25] Lyubenova A, Neupane D, Levis B, Wu Y, Sun Y, He C (2020). Depression prevalence based on the Edinburgh Postnatal Depression Scale compared to Structured Clinical Interview for DSM disorders classification: systematic review and individual participant data meta-analysis. Int J Methods Psychiatr Res.

[CR26] McCabe JE, Wickberg B, Deberg J, Davila RC, Segre LS (2021) Listening Visits for maternal depression: a meta-analysis. Arch Womens Ment Health. Jan 15. 10.1007/s00737-020-01101-4. Epub ahead of print 10.1007/s00737-020-01101-4 33452571

[CR27] Matthey A, Fisher J, Rowe H (2013). Using the Edinburgh postnatal depression scale to screen for anxiety disorders: conceptual and methodological considerations. J Affect Disord.

[CR28] Nagle U, Farrelly M (2018). Women’s views and experiences of having their mental health needs considered in the perinatal period. Midwifery.

[CR29] Nath S, Lewis L, Bick D, Demilew J, Howard L (2021). Mental health and fear of childbirth: a cohort study of women in an inner-city materanity service. Birth.

[CR30] Nilsson C, Hessman E, Sjöblom H, Dencker A, Jangsten E, Mollberg M (2018). Definitions, measurements and prevalence of fear of childbirth: a systematic review. BMC Pregnancy Childbirth.

[CR31] Okagbue HI, Adamu PI, Bishop SA, Oguntunde PE, Opanuga AA, Akhmetshin EM (2019). Systematic review of prevalence of antepartum depression during the trimesters of pregnancy. Open Access Maced J Med Sci.

[CR32] O'Connell MA, Leahy-Warren P, Khashan AS, Kenny LC, O'Neill SM (2017). Worldwide prevalence of tocophobia in pregnant women: systematic review and meta-analysis. Acta Obstet Gynecol Scand.

[CR33] O’Connor EA, Senger CA, Henninger M, Gaynes BN, Coppola E, Soulsby WM (2019) Interventions to prevent perinatal depression: a systematic evidence review for the U.S. preventive services task force. Evidence synthesis. AHRQ publication no 18–05243-EF-1, vol. no. 172. Rockville, MD: Agency for Healthcare Research and Quality; 2019 30807060

[CR34] Olieman R, Siemonsma F, Bartens M, Garthus-Niegel S, Scheele F, Honig A (2017). The effect of an elective cesarean section on maternal request on peripartum anxiety and depression in women with childbirth fear: a systematic review. BMC Pregnancy Childbirth.

[CR35] Pallant J (2013) SPSS Survival Manual, 5th edition. Allen & Unwin, Sydney. ISBN 978-1-74331-400-5

[CR36] Public Health Agency of Sweden. Reduced mental well-being (2018). https://www.folkhalsomyndigheten.se/livsvillkor-levnadsvanor/psykisk-halsa-och-suicidprevention/statistik-psykisk-halsa/. Accessed 2021-08-02

[CR37] Region Stockholm (2020) Screening for fear of birth. Retrieved from: https://vardgivarguiden.se/kunskapsstod/bmm-bvc-forlossning/barnmorskemottagning/riktlinjer/forlossningsradsla/. Accessed 2021-08-15

[CR38] Rondung E, Ekdahl J, Rubertsson C, Hildingsson I, Sundin Ö (2018). Heterogenity in childbirth fear or anxiety. Scand J Psychol.

[CR39] Rondung E, Ternström E, Hildingsson I, Haines H, Sundin Ö, Ekdahl J (2018). Comparing Internet-based cognitive behavioral therapy with standard care for women with fear of birth: Randomized controlled trial. JMIR Mental Health.

[CR40] Rubertsson C, Waldenström U, Wickberg B (2003). Depressive mood in early pregnancy: prevalence and women at risk in a national Swedish sample. J Reprod Infant Psychol.

[CR41] Rubertsson C, Wickberg B, Gustavsson P, Rådestad I (2005). Depressive symptoms in early pregnancy, two months and one year postpartum-prevalence and psychosocial risk factors in a national Swedish sample. Arch Womens Ment Health.

[CR42] Rubertsson C, Waldenström U, Wickberg B, Rådestad I, Hildingsson I (2005). Depressive mood in early pregnancy and postpartum: prevalence and women at risk in a national Swedish sample. JRIP.

[CR43] Rubertsson C, Börjesson K, Berglund A, Josefsson A, Sydsjö G (2011). The Swedish validation of Edinburgh postnatal depression scale (EPDS) during pregnancy. Nord J Psychiatr.

[CR44] Santos H, Tan X, Salomon R (2017). Heterogeneity in perinatal depression: how far have we come? A systematic review. Arch Womens Ment Health.

[CR45] SBU. Swedish Agency for Health Technology Assessment and Assessment of Social Services. Fear of birth, depression and anxiety during pregnancy. A systematic review and assessment of medical, economic, social and ethical aspects. An HTA Report (2021) [In Swe: Förlossningsrädsla, depression och ångest under graviditet]. Stockholm: Statens beredning för medicinsk och social utvärdering (SBU); 2021. SBU-report nr 322. ISBN 978–91–88437–66–2

[CR46] SFOG & SBF. Swedish Association of Obstetricians and Gyneacologists and the Swedish Association of Midwives (2016) Antenatal care, Sexual and Reproductive Health. [In Swe: Mödrahälsovrd, sexuell poch reproduktiv hälsa] Report no 59, updated 2016. Retrieved from: https://www.sfog.se/natupplaga/ARG76web43658b6c2-849e-47ab-99fa-52e8ac993b7d.pdf. Accessed 2021-08-07

[CR47] Storksen HT, Eberhard-Gran M, Garthus-Niegel S, Eskild A (2012). Fear of childbirth; the relation to anxiety and depression. Acta Obstet Gynecol Scand.

[CR48] Swedish National Board of Health and Welfare (2020). National recommendations for depression and anxiety disorders. ISBN 978–91–7555–537–9 Retrieved from: https://www.socialstyrelsen.se/globalassets/sharepoint-dokument/artikelkatalog/nationella-riktlinjer/2020-9-6936.pdf. Accessed 2021-08-15

[CR49] Wickberg B, Hwang CP (1996). The Edinburgh Postnatal Depression Scale: validation on a Swedish community sample. Acta Psychiatr Scand.

[CR50] Wikman A, Axfors C, Iliadis SI, Cox J, Fransson E, Skalkidou A (2020). Characteristics of women with different perinatal depression trajectories. J Neuro Res.

[CR51] Woody CA, Ferrari AJ, Siskind DJ, Whiteford HA, Harris MG (2017). A systematic review and meta-regression of the prevalence and incidence of perinatal depression. J Affect Disord.

